# A Scoping Review on Shoulder Injuries of Wheelchair Tennis Players: Potential Risk-Factors and Musculoskeletal Adaptations

**DOI:** 10.3389/fresc.2022.862233

**Published:** 2022-04-07

**Authors:** Laura Mayrhuber, Thomas Rietveld, Wiebe de Vries, Lucas H. V. van der Woude, Sonja de Groot, Riemer J. K. Vegter

**Affiliations:** ^1^Center for Human Movement Sciences, University Medical Center Groningen, University of Groningen, Groningen, Netherlands; ^2^Swiss Paraplegic Research, Nottwil, Switzerland; ^3^School of Sport Exercise & Health Sciences, Peter Harrison Centre for Disability Sport, Loughborough University, Loughborough, United Kingdom; ^4^Center for Rehabilitation, University Medical Center Groningen, University of Groningen, Groningen, Netherlands; ^5^Amsterdam Rehabilitation Research Center Reade, Amsterdam, Netherlands; ^6^Department of Human Movement Sciences, Faculty of Behavioral and Movement Sciences, Vrije Universiteit, Amsterdam, Netherlands

**Keywords:** wheelchairs, shoulder injuries, physical activity, wheelchair tennis, adapted sports

## Abstract

Wheelchair tennis players are prone to develop shoulder injuries, due to the combination of wheelchair propulsion, overhead activities and daily wheelchair activities. A methodical literature search was conducted to identify articles on shoulder complaints in wheelchair tennis, wheelchair sports and tennis. The aims were to identify (1) type of shoulder complaints; (2) possible risk factors for the development of shoulder injuries; (3) musculoskeletal adaptations in the shoulder joint in wheelchair tennis players. Fifteen papers were included in this review, five on wheelchair tennis, three on wheelchair sports and seven on tennis. Type of shoulder complaints were acromioclavicular pathology, osteoarthritic changes, joint effusion and rotator cuff tears. Possible risk factors for the development of shoulder injuries in wheelchair tennis are overhead movements, repetitive activation of the anterior muscle chain and internal rotators, as well as a higher spinal cord injury level. Muscular imbalance with higher values for the internal rotators, increase in external range of motion, decrease in internal range of motion and reduced total arc of motion were the most common proposed musculoskeletal adaptations due to an unbalanced load. These presented risk factors and musculoskeletal adaptations might help researchers, coaches and wheelchair tennis players to prevent shoulder injuries.

## Introduction

Wheelchair sports participation, like wheelchair tennis, is growing in popularity and is a great opportunity for people with disabilities to get physically active (1). Wheelchair users have an elevated risk to develop various diseases due to a restricted mobility and often sedentary lifestyle, therefore, exercise is crucial to maintain health (2–4). Even though sports participation in wheelchair sports has a broad range of positive effects, it also leads to an increase in stressors on the shoulder complex in addition to the loading from daily activities (5, 6). The prevalence of shoulder problems in wheelchair athletes is reported to have a broad range, i.e., from 16% (7) up to 76% (8). This is similar to able-bodied tennis, in which the shoulder is the most common area of injury of the upper extremity (9). Shoulder pain is prevalent in 24% of the elite tennis players (12–19 years old) (10).

Becoming wheelchair dependent changes the role of the shoulder complex, from providing a great range of motion (ROM) to perform small and detailed movements, into the main source of power for mobility in daily life (11). The motion sequence of wheelchair propulsion itself puts relatively low internal joint forces on the shoulder during regular wheelchair propulsion (12, 13). However, the high frequency of performing the movement in addition to the high shoulder load during specific daily activities, such as transfers in and out of the wheelchair, result in a high exposure to the shoulder joint (12). Changes in the role of the shoulder complex, which require an increased force generation of the upper extremity might lead to imbalances of the muscular system and impact the positioning of the scapula in respect to the humerus as well as both in respect to the thorax (14). Altered conditions in the shoulder joint favor an impingement within the subacromial space and a greater abrasion of the joint (6, 15).

Wheelchair tennis is the most popular adapted racket sport but it involves a high incidence of shoulder complaints (16–19). In wheelchair tennis, the tennis racket is an additional constraint during propulsion of the wheelchair since it interferes with the hand/rim interaction (20). With the racket in one hand, which leads to unilateral power losses because of the more difficult coupling to the hand rim, greater forces need to be produced to maintain balanced power production at both sides (21, 22). As in able-bodied tennis, wheelchair tennis players have a repetitive activation of the anterior muscle chain, due to the unidirectional movements of the strokes. Furthermore, a seated position, as is the case in wheelchair tennis, leads to a modified force generation, as well as changes in shoulder alignment and trunk rotations (17, 23, 24). The core stability and sitting position in the wheelchair have a great impact on the shoulder mechanics and, therefore, on the force generation in the serve and ground strokes (23).

Wheelchair dependence and overhead activities in combination with high training intensities increase the already heavy strain on the shoulder and might be a possible risk factor for overuse injuries in wheelchair tennis athletes (22, 25). Injuries to the upper extremity or overuse symptoms not only negatively affect sport performance but also have a tremendous impact on body functions, activity, and participation in daily life (11). Therefore, it is highly important to identify possible causes and aggravating factors and avoid shoulder injuries in wheelchair tennis. The aims of this review are to: (1) identify type of shoulder complaints; (2) potential risk factors for the development of shoulder injuries in wheelchair tennis; (3) investigate potential musculoskeletal adaptations causing shoulder complaints in the shoulder joint in wheelchair tennis. Given the small number of wheelchair tennis papers, an overview will be given from a wheelchair tennis perspective, as well as a broader view from a wheelchair sports and able-bodied tennis perspective. Due to the recency of written reviews by Heyward et al. (22) on shoulder injuries in wheelchair sports and by Kekelekis et al. (26) on shoulder injuries in able-bodied tennis, these two papers were taken as central papers in the respective parts of the current review and extended with additional papers.

## Materials and Methods

### Search Strategy

A methodical search strategy was conducted in October 2020 using the PRISMA checklist (Supplementary Table 1) for Scoping reviews by two independent researchers (LM, TR) to identify relevant published articles on the topic of shoulder complaints in (i) wheelchair tennis, (ii) wheelchair sport and (iii) tennis. In case of discrepancies between authors, articles were discussed between the two researchers. PubMed and Web of Science were used to search for relevant articles. The PubMed search strategy shown below was adapted for the second database Web of Science.

(1) (“Wheelchairs”[Mesh])

(2) (“Sports”[Mesh])

(3) (“Tennis”[Mesh])

(4) (“Shoulder Joint”[Mesh] OR “Upper Extremity”[Mesh] OR “Shoulder”[Mesh] OR “Scapula”[Mesh] OR “Rotator Cuff”[Mesh])

(5) (“Muscle Strength”[Mesh] OR “Pain”[Mesh] OR “Musculoskeletal Pain”[Mesh] OR “Chronic Pain”[Mesh] OR “Shoulder Pain”[Mesh] OR “Wounds and Injuries”[Mesh] OR “Athletic Injuries”[Mesh] OR “Rotator Cuff Injuries”[Mesh] OR “Tendon Injuries”[Mesh] OR “Stress Disorders, Post-Traumatic”[Mesh] OR “Arm Injuries”[Mesh] OR “Shoulder Impingement Syndrome”[Mesh] OR “Shoulder Injuries”[Mesh] OR “Bursitis”[Mesh] OR “Rotator Cuff Tear Arthropathy”[Mesh] OR “Risk”[Mesh] OR “Risk Factors”[Mesh] OR “Health Risk Behaviors”[Mesh] OR “Pathology”[Mesh] OR “Syndrome”[Mesh] OR cause^*^[tiab] OR mechanism^*^[tiab] OR complaint^*^[tiab] OR discomfort^*^[tiab])

Search string – Wheelchair tennis: (1), (3), (4) and (5)

Search string – Wheelchair sports: (1), (2), (4) and (5)

Search string – Tennis: (3), (4) and (5)

Articles from the database search were first checked for duplicates, secondly the titles and abstracts were screened. Thirdly, the full text of the remaining articles was assessed and included if criteria were met.

#### Inclusion Criteria

Articles in the English language that incorporated some type of shoulder complaint or assessment either in wheelchair sports, tennis or a combination of the two.

#### Exclusion Criteria

Papers from all categories (wheelchair tennis, wheelchair sports, tennis) were excluded if they had a treatment/ intervention program, an assessment was evaluated/tested and when it was an epidemiological study. For the able-bodied tennis and wheelchair sport papers, articles were also excluded when pain in the shoulder joint was not reported. This was not an exclusion criterion for the wheelchair tennis papers, due to the scarcity of available literature.

### Data Extraction and Quality Assessment

Quality assessment was also performed by two independent researchers (LM, TR) for all included articles and was performed with a checklist of Webster et al. (27), adapted by Heyward et al. (22). This checklist was chosen because there is no standardized checklist available for this type of study. For each question a score of 1 was given for an “adequate” or “yes” response, a score of 0.5 was given for a “partial” or “limited” response; and a score of 0 was awarded for a “no”, “not stated” or “inadequate” response. A maximum score of 8 was possible. There were no minimum criteria set due to the limited number of papers that were included in the study.

### Definitions of Risk Factors and Musculoskeletal Adaptations

**Figure 1:**
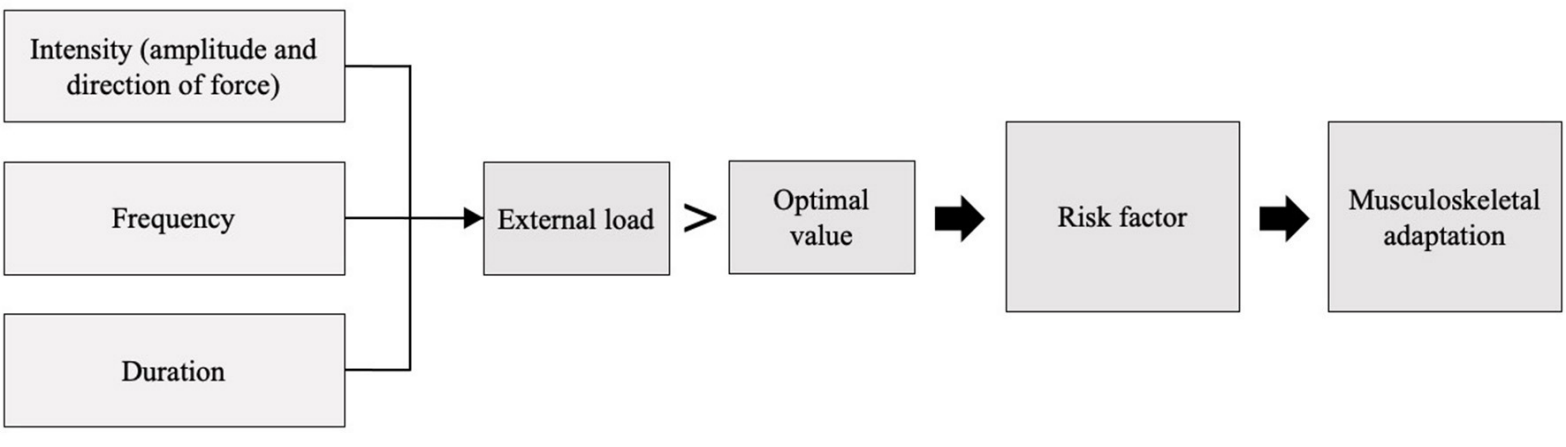
Conceptual model of risk factors and musculoskeletal adaptations for shoulder injuries, based on the model of Hoozemans et al. (28).

Risk factors for complaints in this review were defined based on Hoozemans et al. (28) in which “external load” was defined using three factors: intensity, frequency and duration (Figure 1). The risk for complaints occurs if the value of one of these three factors or the combination of the factors deviates from their optimal value (28, 29). Musculoskeletal adaptions are caused by the risk factors and lead to unfavorable biomechanical conditions in the shoulder complex. An example of a risk factor could be an increased internal rotation balance ratio, due to greater activation of the anterior muscles and repetitive movements. The musculoskeletal adaptation that occurs could be a muscular imbalance. Due to the limited research in the topic, statistically proven risk factors as well as proposed risk factors were included in this review.

## Results

**Figure 2:**
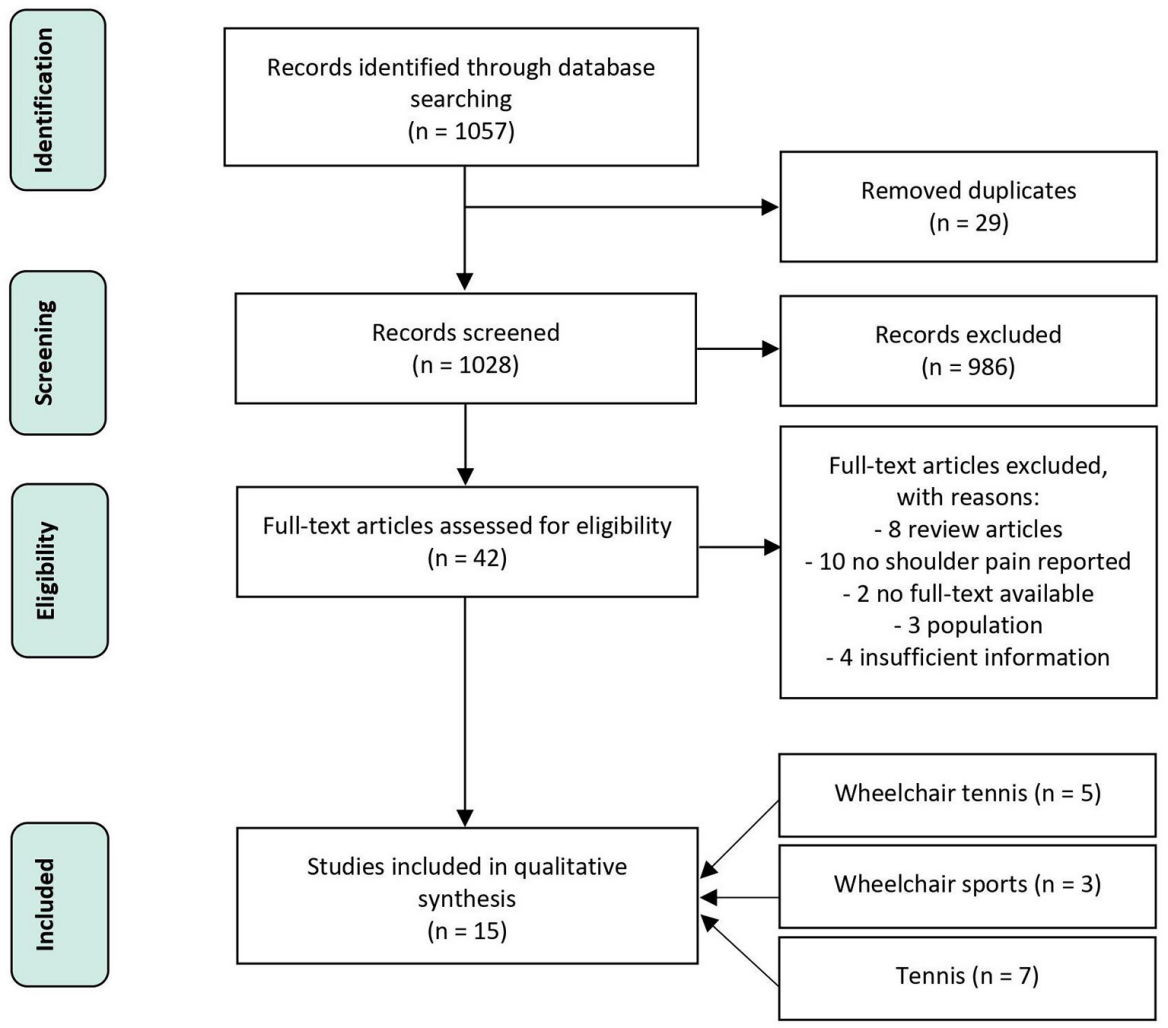
Flow chart describing the selection process included.

A flow chart of the selection process is shown in Figure 2. Five papers were included regarding wheelchair tennis. For wheelchair sports, an interpretation of 13 papers of the review of Heyward et al. (22) will be given, with an additional three papers selected for the current review. For the tennis papers, an interpretation of 23 papers of the review of Kekelekis et al. (26) will be given, with an additional seven papers for the current review.

In 12 of the 15 included articles shoulder problems or a history of shoulder problems were reported, of which eight included clinical testing of the shoulder complaint. A wide variety in screening of indicators for shoulder complaints were reported. Radiographic analysis was used in three articles (18, 30, 31), a strength test in six (30, 32–36), a kinematic analysis in three (5, 24, 37), a kinetic analysis in two (38, 39), the Wheelchair User's Shoulder Pain Index (WUSPI) in two (5, 31), visual analogue scale (VAS) in two (33, 34), range of motion (ROM) measurement in four (32–34, 40) and the scapular resting position (25), perceived function (25), serve speed (34) and post impact ball velocity (39) in one of the articles.

### Quality of the Evidence

The results of this review should be viewed with consideration to the level of evidence (Supplementary Table 2). The quality of the articles in the review of Heyward et al. (22) were checked using the same checklist and ranged from low (3) to good (7), with a mean of around 4.5. Especially inclusion/exclusion criteria, reliability and validity were poorly described across papers. The quality of the articles in the review of Kekelekis et al. (26) were checked using a different, extensive checklist by Downs and Black (41). Due to the great number of subcategories and their specificity, the overall score average was low to moderate, with especially the internal validity lacking.

Of all 15 included papers in the current review, eight described the participants characteristics adequately (5, 18, 24, 31–34, 36). Six papers fully described inclusion and exclusion criteria (5, 25, 30, 33, 34, 40) and 13 described the limitations of the study (5, 18, 25, 30–35, 37–40). The key variables, pain, strength and injuries were measured adequately in seven of the 15 papers (5, 18, 24, 32, 34, 37, 39). Overall, the validity and reliability of the used assessments had limited description. In six of the included studies the reliability (5, 24, 30, 32, 33, 40) and in seven the validity (5, 24, 30, 31, 33, 39, 40) were adequately described. Only two papers (25, 32) adequately discussed the external validity of the results.

### Type of Shoulder Complaints

#### Wheelchair Tennis

An overview of the included papers can be seen in Table 1. In the wheelchair tennis papers, two (5, 18) of the five papers reported shoulder complaints by the participants. Causes of complaints in wheelchair tennis were acromioclavicular pathology in the dominant shoulder, osteoarthritic changes, joint effusion and rotator cuff tears (18) in the dominant as well as the nondominant shoulder, most commonly in the supraspinatus tendon. The paper of Warner et al. (5) reported two participants with previously experienced pain due to shoulder impingement and one participant with subacromial pain syndrome.

**Table 1:** Overview of articles describing type of shoulder complaints, proposed risk factors and musculoskeletal adaptations in wheelchair tennis.

**References**	**QAS (0–8)**	**Sport (N)**	**Disability types**	**M/F**	**Age (mean)**	**Cases shoulder pain/injury**	**Type of complaint**	**Objective measure**	**Clinical testing**	**Activity level**	**Sport activity/TSI (years)**	**Proposed risk factor**	**Musculo skeletal adaptation**
Bernard et al. (36)	3	WRa/WT (21), ABT (15)	12 high lesions, 9 low lesions	36/0	27	X	X	Strength test	X	X	X/13	Level of SCI	Muscular imbalance
Jeon et al. (18)	5	WT (33)	Paraplegic	26/7	36	23	Pain, AC pathology, rotator cuff tears, biceps tendon pathology, sub-acromial/ deltoid effusion	Radiographic analysis	Yes	4–7 h/day	5–15/6–20	Overuse, repetitive impingement positioning	Scapula dyskinesis
Moon et al. (35)	2.5	WT (12)	10 SCI, 1 amputee, 1 other	X	33	X	X	Strength test	X	X	7/X	X	Muscular imbalance
Reid et al. (24)	3.5	WT (2)	1 L1, 1 Incomplete T10 SCI	2/0	X	X	X	Kinematic analysis	X	X	X	Reduced shoulder joint loading	X
Warner et al. (5)	7.5	WT (11)	X	8/3	27	1	Previously experienced pain	Kinematic analysis, WUSPI	Yes	18 h/week	X / 15	X	Scapula posterior tilt & external rotation

*QAS, quality assessment score; TSI, time since injury; AC, acromio-clavicular; WT, wheelchair tennis; WRa, wheelchair racing; ABT, able-bodied tennis; SCI, spinal cord injury; WUSPI, wheelchair user shoulder pain index*.

#### A Broader View From Wheelchair Sports and Able-Bodied Tennis

Pain was reported as the most frequent shoulder complaint in the review of Heyward et al. (22) and the three additional selected papers (25, 31, 37) (Table 2). Other shoulder problems included rotator cuff tears, rotator cuff impingement, acromion-clavicular and bicep tendon pathology, subdeltoid and subacromial effusion, as well as non-specific shoulder issues (22). In one of the additional included papers (31) tendinopathy and bursitis were listed as other shoulder complaints. A history of shoulder problems in the selected able-bodied tennis papers and the review of Kekelekis et al. (26) were tendinosis (30), general pain (33, 34, 40, 42), rotator cuff tears or tendinopathy (38, 39, 42, 43), osteoarthritic changes (44) and labral lesion or tears (38, 42, 43).

**Table 2:** Overview of articles describing type of shoulder complaints, proposed risk factors and musculoskeletal adaptations in wheelchair sports.

**References**	**QAS (0-8)**	**Sport (N)**	**Disability types**	**M/F**	**Age (mean)**	**Cases shoulder pain/injury**	**Type of complaint**	**Objective measure (s)**	**Clinical testing**	**Activity level**	**Sport activity/TSI (years)**	**Proposed risk factor**	**Musculo skeletal adaptation**
Aytar et al. (25)	5	Amputee soccer, WB WTT (63)	29 amputees, 10 poliomyelitis, 4 spina bifida, 6 SCI, 14 others	55/8	24	X	General pain in the shoulder	Scapular resting position, pain, perceived function	X	X	6 months /X	X	Abnormal scapula resting position
Mason et al. (37)	4	WR (10)	6 HP & 4 LP players	X	34	5	General pain in the shoulder	Kinematic analysis	X	X	X/14	X	Rotated scapula
You et al. (31)	4.5	WTT (19), WAR (16)	31 SCI, 3 amputees	24/11	47	X	Tendinopathy, bursitis	WUSPI, Radiographic analysis	Yes	24,8 h/week	15/25	Overuse, high torques on shoulder	X

*QAS, quality assessment score; TSI, time since injury; WB, wheelchair basketball; WR, wheelchair rugby; WTT, wheelchair table tennis, WAR, wheelchair archery; SCI, spinal cord injury; WUSPI, wheelchair user shoulder pain index; HP, High-point; LP, Low-point*.

### Risk Factors

**Figure 3:**
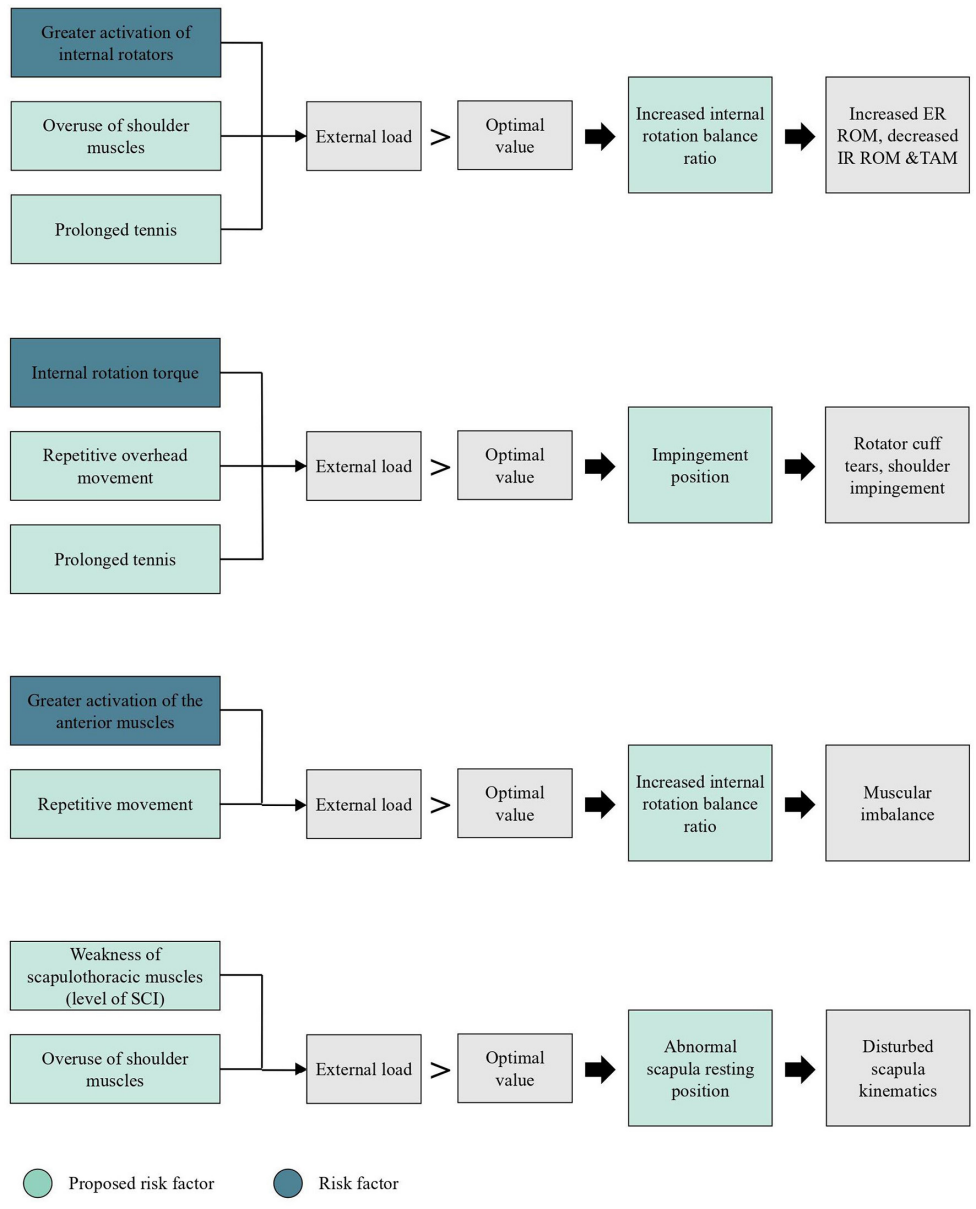
Schematic representation of (potential) risk factors and musculoskeletal adaptations for shoulder injuries in wheelchair tennis, based on the conceptual model of Hoozemans et al. (28). ER = external rotation, IR = internal rotation, ROM = range of motion, TAM = total arc of motion, SCI = spinal cord injury.

The interpretation of the possible relationships between risk factors and musculoskeletal adaptions are schematically represented using the previous defined model of Hoozemans (Figure 3). Due to the low number of articles in wheelchair tennis describing risk factors and musculoskeletal adaptations, a broader view from wheelchair sports and able-bodied tennis is presented as well. Firstly, the risk factors will be described, secondly the musculoskeletal adaptation. These summarizing results will be further interpreted in the discussion part.

#### Wheelchair Tennis

A proposed risk factor for shoulder problems in wheelchair tennis, especially in the dominant shoulder, is overuse, caused by wheelchair propulsion, transfers in and out of the wheelchair and playing tennis (18). The repetitive impingement positioning during the play can lead to rotator cuff tears, especially the supraspinatus muscle, and high compressive forces on the acromioclavicular joint (18). The risk for overuse increases when the internal rotation balance ratio is higher compared to the normal range (35). Another proposed risk factor is the level of spinal cord injury (SCI) (36). Higher values of torque and power for internal and external muscles were observed in athletes with a low level SCI (T11-L3) in comparison with athletes with a higher level SCI (T5-T8) (36). The level of lesion does not necessarily influence the rotator balance ratio in the shoulder by the activation of internal rotator muscles but by the participation of the external rotators (36). Age, training time per day, duration of wheelchair usage and wheelchair tennis career did not present as risk factors (18).

#### A Broader View From Wheelchair Sports and Able-Bodied Tennis

Participating in wheelchair sports bears several risk factors for shoulder problems, which are multifactorial (22). Proposed risk factors were overuse of the shoulder muscles and less trunk control. Performing overhead sports in a wheelchair increases the risk for rotator cuff tears due to overhead motion and the recurrent microtrauma (8). Repetitive shoulder movements with a great internal rotation torque can lead to the occurrence of a mechanical shoulder impingement and are a proposed risk factor for shoulder overuse injuries (31). No associations were observed between age, wheelchair usage duration, training load and the amount of pain due to shoulder problems (31).

Prolonged tennis exposure was identified as the most common proposed risk factor for able-bodied tennis players in the review of Kekelekis et al. (26), due to its negative effect on muscle performance, serve maximal angular velocities and joint kinetics (26, 45). Furthermore, skill level and technique of the player were identified as risk factors. Less shoulder joint load with a lower risk for the development of injuries was observed in professional tennis players (43). Additional risk factors were a prolonged abduction during the external rotation phase of the serve (46) and scapula dyskinesia (47). Proposed risk factors were a stiffer racket (48), racket with a higher polar moment of inertia (49) and previous injuries (46, 50).

The additional selected papers in this review showed that repetitive overhead movements (42) and overuse due to rigorous training schedules (32) seem to be related to shoulder injuries (Table 3). During the serve and smash, the dominant arm is in an abducted position with full external rotation and extension which leads to structural lesions of the rotator cuff and superior labral lesions (42). The overhead motion causes repetitive microtraumas to the capsule and a posterior capsule tightness in tennis players with shoulder pain can be observed (33). Additionally, serve variations like the waiter's serve (39) as well as improper techniques (38) are risk factors for the development of overuse injuries, since alterations in timing of trunk and shoulder rotation in the serve can lead to higher shoulder joint loads. Lastly, a strong upper trapezius in both sides (32) was listed as a proposed risk factor for the development of shoulder injuries in tennis players.

**Table 3:** Overview of articles describing type of shoulder complaints, proposed risk factors and musculoskeletal adaptations in able-bodied tennis.

**References**	**QAS (0–8)**	**Sport (n)**	**M/F**	**Age (mean)**	**Cases shoulder pain/injury**	**Type of complaint**	**Objective Measure**	**Clinical testing**	**Activity level**	**Sport activity/TSI (years)**	**Proposed risk factor**	**Musculo skeletal adaptation**
Gillet et al. (32)	6.5	ABT (91)	91/0	11	30	History of shoulder problems	Strength test, ROM	Yes	11h/week	6/None	X	Muscular imbalance, increased GH ROM
Johansson et al. (30)	6.5	ABT (35)	15/20	17	X	Tendinosis	Radiographic analysis, strength test	Yes	12–20 h/week	X/None	X	Larger infraspinatus & teres minor
Marcondes et al. (33)	8	ABT (49)	49/0	26	27	Pain in the shoulder	VAS, ROM, strength test	Yes	8–12 h/week	8/None	ER strength deficit	Posterior capsule tightness, IR deficit, ER gain
Martin et al. (38)	4	ABT (20)	20/0	25	6	SLAP lesion, RC tendinopathy, labral tears	Kinetic values, post impact ball velocity	X	X	X	Timing trunk/shoulder rotation in serve, lower ball velocity, high joint kinetics^*^	X
Moreno-Perez et al. (40)	6.5	ABT (47)	43/0	23	19	History of shoulder pain	ROM	X	X	16/None	X	Decreased GH IR & TAM
Moreno-Pérez et al. (34)	5	ABT (58)	58/0	21	20	History of shoulder pain	ROM, serve speed, strength test, VAS	Yes	17 h/week	13/None	X	Muscular imbalance, increased ER ROM, reduced IR ROM
Touzard et al. (39)	4.5	ABT (18)	18/0	14	17	Shoulder tendinopathy	Kinetic analysis, post-impact ball velocity,	Yes	X	X	Waiters serve posture, higher upper limb kinetics^*^	X

*QAS, quality assessment score; TSI, time since injury; ABT, able-bodied tennis; GH, Glenohumeral; ROM, range of motion; ER, external rotation; IR, internal rotation; TAM, Total arc of motion; VAS, visual analogue scale; SLAP, superior labral tear from anterior to posterior; RC, rotator cuff*.

* *Statistically proven risk factors*.

### Musculoskeletal Adaptations

#### Wheelchair Tennis

In wheelchair tennis, the supposed musculoskeletal adaptations in shoulder problems are multifactorial. Three of the five papers (18, 35, 36) mentioned a muscular imbalance as alteration in the shoulder girdle, but only one (18) connected it with the occurrence of shoulder problems. Two papers (35, 36) described a muscular imbalance with a higher extension than flexion strength and higher values for internal than external rotator muscles, especially on the dominant side. Differences between the dominant and non-dominant side for scapula posterior tilt were observed, with a more posteriorly tilted scapula on the dominant side (5). The upwardly rotated scapula of the dominant arm in wheelchair tennis players was higher compared to able-bodied participants with shoulder impingement (5).

#### A Broader View From Wheelchair Sports and Able-Bodied Tennis

Musculoskeletal adaptations associated with shoulder pain in wheelchair sports were difficult to identify. In the review of Heyward et al. (22) it was suggested that shoulder pain was connected to weaknesses in the internal/external rotation, as well as adduction of the shoulder. In one of the additional included papers (25) it was discussed that a weakness of scapula thoracic muscles due to participation in wheelchair sports potentially leads to an abnormal positioning of the scapula. As a consequence, disturbances in the scapula humeral rhythm and general shoulder dysfunction might be observed (25). Scapula position in bilateral shoulder pain in symptomatic individuals had less upward rotation than symptomatic individuals with unilateral pain (37). During the push phase, the scapula moves towards a more internally, upwardly rotated and less anterior position. During the recovery phase the scapula maintained an upward rotated position (37).

Muscular imbalance in the shoulder joint was the most frequent proposed musculoskeletal adaptations in shoulder problems in able-bodied tennis players (30, 32, 34). The studies describe an unbalanced ratio between internal and external rotators in tennis players, especially in the dominant arm. Increases in internal rotators strength are favored due to the demand during tennis strokes (30, 32, 34). A deficit in external rotation strength in the dominant arm in tennis players with shoulder pain has been observed in the study of Marcondes et al. (33). With an imbalance of the muscular system, a change of ROM often takes place, which can be associated with shoulder problems. Four papers (32–34, 40) describe an increase in external ROM, a decrease in internal ROM and a reduced total arc of motion (TAM) in the glenohumeral joint of the dominant arm of tennis players with a history of shoulder pain. The TAM, is defined as the sum of internal rotation ROM and external rotation ROM (32).

## Discussion

The aim of the current review was to identify type of shoulder complaints and potential risk factors for the development of shoulder injuries in wheelchair tennis and investigate potential musculoskeletal adaptations in the shoulder joint in wheelchair tennis players. In the course of this review, risk factors and musculoskeletal adaptations in wheelchair tennis, wheelchair sports and able-bodied tennis were presented (Figure 3). There was a scarcity of literature in all three areas, but by connecting available literature, implications for future research and practice were derived.

Overhead activity with the shoulder joint in an impingement position was proposed as a risk factor for shoulder problems in wheelchair tennis (18), wheelchair sports with an overhead movement (8) and able-bodied tennis (42). Overhead activities, like the service or smash in tennis, repeatedly decrease the subacromial cavity by an elevation of the upper arm and lead to an impingement position (42). The supraspinatus tendon passes laterally beneath the cover of the acromion and the bursa subacromialis in the subacromial cavity, therefore it can be damaged due to the repetitive mechanical impingement (51). That could explain the high prevalence of supraspinatus pathology and bursitis in the dominant arm in athletes performing overhead activities (8, 18, 31, 42).

In tennis players with a history of shoulder problems, a reduced glenohumeral TAM was observed (32). Tennis players appear to evolve an increase in external ROM due to osseus alterations, a decrease in internal ROM due to stiffening of the posterior capsule and a loss of TAM in the dominant arm (32, 34). A loss of internal ROM and TAM in the dominant arm compared to the non-dominant arm is a common adaptation in shoulder injuries (32, 34). The rotator cuff muscles have to compensate for the integrity of the shoulder if the ROM and flexibility increases which could then lead to an overuse of the rotator cuff muscles (32, 34).

The combination of being wheelchair-bound and being an overhead athlete can cause alterations in the position of the shoulder joint and scapula which leads to unfavorable biomechanical conditions in the shoulder complex. Wheelchair tennis consists of short intermittent sprints, that demand a constant acceleration and deceleration with changes in direction, as well as the generation of powerful serves and groundstrokes (11, 22). Due to the seated position and lower ball velocities during the serve, wheelchair tennis players reported less load on the shoulder compared to able-bodied tennis players (24). Wheelchair propulsion as well as playing tennis lead to an unbalanced ratio in the dominant arm in tennis players between internal/external rotators due to a high demand of internal rotators during strokes (34, 36). Comparing wheelchair tennis players with able-bodied tennis players, even higher values for internal rotation were observed (36), which suggests a greater muscular imbalance in wheelchair tennis players.

A higher risk of muscular imbalance and shoulder problems seems to occur in wheelchair tennis athletes who have a higher level of SCI and, as a consequence, less trunk control (36). This is in line with the findings of Heyward et al. (22) in which wheelchair athletes with low trunk control had more shoulder complaints compared to athletes with high trunk control. The lack of muscular control and stabilization in the trunk limits the power generation in the kinematic chain (7). Therefore, the upper body has to compensate for the lack of power, which can overload the shoulder joint and increases the stress on the joint (22). In addition, Bernard et al. (36) suggest that a higher level of SCI influences the internal and external rotator ratio by the preferential development of flexor, internal rotator, and adductor muscles. A muscular imbalance oftentimes alters the scapula position to a more upward and internal rotated position (37). In this abnormal position, the impingement within the subacromial space in the shoulder joint is favored and a greater abrasion of the joint occurs, which is suggested to be one of the reasons for shoulder injuries (5, 35).

Given the above-stated factors, wheelchair tennis players are expected to be prone to develop a muscular imbalance which leads to alterations in the joint positioning. This is supported by a study of Aytar et al. (25) that showed that a high percentage of abnormal scapular resting positions was prevalent in wheelchair sports players, which was associated with pain as well as bad perceived shoulder function. In contrary to this hypothesis, Warner et al. (5) reported that the scapula was more posterior tilted and externally rotated on the dominant than the non-dominant side and only one of the wheelchair tennis players reported pain. Postural abnormalities of the scapula, with a protraction of the scapula are associated with decreasing the subacromial space and the prevalence of shoulder impingement (52). The absence of shoulder pain might be related to the posterior tilt of the scapula. A reduced upward rotation, external rotation and posterior tilt of the scapula are increasing the sub-acromial space, which leads to less abrasion in the shoulder joint (52). The connection between an upwardly rotated scapula and a higher prevalence of pain, was also described by Warner et al. (5). It was suggested that an absence of shoulder pain occurred due to a posterior tilted and externally rotated scapula in the dominant arm. The low prevalence of shoulder pain reported in this sample may be explained by a protective benefit due to a specific training program or sports participation, that prevents a protraction and internal rotation of the scapula (5).

### Future Research

Further research should be directed toward more specific wheelchair tennis research focused on the load of the shoulder, risk factors and musculoskeletal adaptations. Shoulder load was never assessed in wheelchair tennis, only the influence of the racket and a different hand rim were investigated (20, 21, 53, 54). First the influence of the racket on shoulder load should be investigated, afterwards wheelchair tennis players with and without shoulder complaints could be compared to identify differences. Further investigation of identified risk factors and musculoskeletal adaptations in the course of this review, such as muscular imbalance and alterations in ROM, can give valuable insight for the development of preventive training and exercise programs for wheelchair tennis players.

### Limitations

Overall, the lack of publications and research in the wheelchair tennis field brought a limited number of papers out of the literature search that investigated shoulder joint injuries in wheelchair tennis. Due to the lack of high-quality literature on wheelchair tennis to be included in this review, it was necessary to combine it with papers about shoulder complaints in other wheelchair sports and able-bodied tennis. This review is a first attempt to gain insight into potential risk factors for shoulder injuries in wheelchair tennis and their musculoskeletal adaptations by comparing and connecting the available information with outcomes of tennis and other wheelchair sports papers.

Additionally, it is important to mention that the included articles about wheelchair sports in general had a relatively low number of participants, which is a common problem in wheelchair sport literature (55). Several papers did not directly investigate risk factors and musculoskeletal adaptations but proposed multiple potential reasons based on their findings, which makes wheelchair tennis focused research even more important. Furthermore, the studies oftentimes did not specify which type of shoulder complaint was the cause and differentiated in the objective measurement tools, which made it challenging to compare the outcomes and draw conclusions.

## Conclusion

Risk factors and musculoskeletal adaptations in wheelchair tennis can only be described from a broader wheelchair sports and tennis perspective. Possible risk factors for the development of shoulder injuries in wheelchair tennis are overhead movements, repetitive activation of the anterior muscle chain and internal rotators, as well as a higher SCI level. Muscular imbalance with higher values for the internal rotators, increase in external ROM, decreased internal ROM and reduced TAM were the most common proposed musculoskeletal adaptations due to an unbalanced load. In the future, these risk factors and musculoskeletal adaptations should be investigated in a more wheelchair tennis focused research.

## Author Contributions

LM, TR, and RV: conceptualization, investigation, and methodology. LM and TR: formal analysis and writing—original draft. RV, LvdW, SdG, and WdV: supervision. LM, TR, RV, LvdW, SdG, and WdV: writing—review & editing. All authors contributed to the article and approved the submitted version.

## Conflict of Interest

The authors declare that the research was conducted in the absence of any commercial or financial relationships that could be construed as a potential conflict of interest.

## Publisher's Note

All claims expressed in this article are solely those of the authors and do not necessarily represent those of their affiliated organizations, or those of the publisher, the editors and the reviewers. Any product that may be evaluated in this article, or claim that may be made by its manufacturer, is not guaranteed or endorsed by the publisher.
