# Supplementary material for: A Scoping Review on Shoulder Injuries of Wheelchair Tennis Players: Potential Risk-Factors and Musculoskeletal Adaptations
Source: Front Rehabil Sci. 2022 Apr 7;3:862233. doi: 10.3389/fresc.2022.862233 (PMC9397987; doi:10.3389/fresc.2022.862233)
Supplement: Supplementary file 2 [file Table_2.DOCX]

**S2 Table: Quality assessment with an adapted version of a checklist developed by Webster et al.** [29]

|  | **1. Participants characteristics** | **2. Inclusion/ exclusion criteria** | **3.Was the design appropriate to the research question?** | **4. Were key dependent variables measured?** | **5. Psycho- metric properties (reliability)** | **6. Activity psycho- metric properties (validity)** | **7. Was the external validity of the results discussed?** | **8. Were the limitations of the study described?** | **Total Score** |
| --- | --- | --- | --- | --- | --- | --- | --- | --- | --- |
| **Bernard et al. (2004)** [37] | ++ | +- | ++ | +- | -- | -- | +- | -- | 3.5/8 |
| **Jeon et al. (2010)** [15] | ++ | +- | ++ | ++ | -- | -- | +- | ++ | 5/8 |
| **Moon et al. (2013)** [22] | +- | -- | +- | +- | -- | -- | -- | ++ | 2.5/8 |
| **Reid et al. (2007)** [21] | ++ | -- | +- | ++ | ++ | ++ | -- | -- | 4.5/8 |
| **Warner et al. (2018)** [5] | ++ | ++ | ++ | ++ | ++ | ++ | +- | ++ | 7.5/8 |
| **Aytar et al. (2015)** [28] | +- | ++ | +- | +- | +- | -- | ++ | ++ | 5/8 |
| **Mason et al. (2018)** [38] | +- | +- | ++ | ++ | +- | -- | -- | ++ | 4.5/8 |
| **You et al. (2016)** [33] | ++ | +- | +- | +- | -- | ++ | -- | ++ | 4.5/8 |
| **Gillet et al. (2018)** [34] | ++ | -- | ++ | ++ | ++ | +- | ++ | ++ | 6.5/8 |
| **Johansson et al. (2015)** [32] | +- | ++ | ++ | +- | ++ | ++ | +- | ++ | 6.5/8 |
| **Marcondes et al. (2013)** [35] | ++ | ++ | ++ | +- | ++ | ++ | +- | ++ | 7/8 |
| **Martin et al. (2014)** [39] | +- | -- | ++ | +- | +- | +- | +- | ++ | 4/8 |
| **Moreno-Perez et al. (2015)** [41] | +- | ++ | ++ | +- | ++ | ++ | +- | ++ | 6,5/8 |
| **Moreno-Perez et al. (2018)** [36] | ++ | ++ | ++ | ++ | -- | -- | -- | ++ | 5/8 |
| **Touzard et al. (2019)** [40] | +- | -- | ++ | ++ | -- | ++ | -- | ++ | 4,5/8 |
